# Supplementary material for: Expression of Steroid Receptor RNA Activator 1 (SRA1) in the Adipose Tissue Is Associated with TLRs and IRFs in Diabesity
Source: Cells. 2022 Dec 11;11(24):4007. doi: 10.3390/cells11244007 (PMC9776802; doi:10.3390/cells11244007)
Supplement: Supplementary file 1 [file cells-11-04007-s001.zip › cells-2046634-supplementary.pdf]

Table S1. Demographic and clinical characteristics of study population. (Published Cells 2021, 10(10), 2602; https://doi.org/10.3390/cells10102602)

	**Total Participants (n=108)**										
	**Non-diabetic (N=55) Median (IQR)**					**Diabetic (N=53) Median (IQR)**					
	**Lean (n = 8)**	**Overweight (n = 19)**	**Obese (n =28)**	**Lean vs. Overweight (p value)**	**Lean vs. Obese (p value)**	**Lean (N=4)**	**Overweight (N=13)**	**Obese (N=36)**	**Lean vs. Overweight (p value)**	**Lean vs. Obese (p value)**	**Non-diabetic vs. Diabetic (p value)**
Age (years)	42.00(36.00-50.25)	42.00(35.00-52.00)	46.50(36.00-59.00)	0.688	0.387	56(49.75-57.75)	54.00(53.00-57.00)	53.00(46.00-58.00)	0.608	0.557	**<0.001**
Weight (kg)	61.00(58.40-77.33)	79(70.2-88)	91.75(84.83-105.70)	**0.004**	**<0.0001**	68.85(57.9-69.75)	77.7(72.5-85.25)	93.50(83.08-99.70)	**0.024**	**0.001**	0.327
Height (cm)	1.66(1.55-1.80)	1.67(1.59-1.76)	1.65(1.55-1.73)	0.906	0.422	1.65(1.538-1.665)	1.66(1.61-1.72)	1.69(1.58-1.73)	0.461	0.223	0.784
BMI (kg/m^2)	23.81(22.07-24.56)	28.43(27.26-29.4)	35.20(31.65-37.23)	**<0.0001**	**<0.0001**	24.99(24.28-25.59)	28.2(27.13-28.87)	33.35(31.47-35.56)	**0.013**	**0.001**	0.417
Waist circumference (cm)	81.00(77.00-97.00)	96(89-101.8)	109.00(99.00-117.00)	**0.014**	**<0.0001**	82(76-87)	98.00(93.00-103.00)	110(104.5-114)	**0.024**	**0.005**	0.052
HIP circumference (cm)	103.00(91.50-105.00)	103.50(97.00-111.5)	120.00(109.50-125.00)	0.324	**<0.0001**	100.00(93.00-106.00)	104.00(101.00-111.00)	113.80(110.00-120.80)	0.273	**0.015**	0.768
WHR	0.82(0.74-0.94)	0.95(0.82-1.02)	0.91(0.81-1.00)	0.105	0.072	0.8172(0.78-0.87)	0.93(0.90-1.01)	0.9571(0.8789-1.018)	**0.016**	**0.021**	0.089
Body fat (%)	26.90(22.30-36.70)	30.9(28.43-37.63)	39.90(36.78-43.63)	0.074	**<0.0001**	34.8(27.70-36.50)	30.7(26.00-37.30)	38(32.2-41.85)	0.938	0.181	0.984
Fasting plasma glucose (mM)	4.90(4.32-5.18)	5.1(4.9-5.43)	5.20(4.75-5.88)	0.27	0.186	7.7(5.65-9.953)	8.72(6.92-11.13)	8.15(6.6-10.03)	0.362	0.471	**<0.0001**
Triglycerides (mmol/L)	0.62(0.43-0.89)	1.3(0.64-1.65)	1.05(0.72-1.53)	**0.014**	**0.042**	0.97(0.47-1.88)	1.79(0.84-2.40)	1.395(1.113-1.818)	0.225	0.131	**<0.001**
Total cholesterol (mmol/L)	5.35(3.88-6.04)	5(4.48-5.42)	4.75(4.16-6.05)	0.769	0.943	4.48(3.8-6.54)	4.09(3.85-5.56)	4.94(4.1-5.745)	0.544	0.701	0.314
HDL cholesterol (mmol/L)	1.59(1.18-2.01)	1.2(1.08-1.36)	1.16(1.02-1.38)	**0.033**	**0.041**	1.14(0.97-1.3)	1.03(0.89-1.31)	1.11(0.95-1.36)	0.671	0.835	0.096
LDL (mmol/L)	3.55(2.33-3.93)	3.1(2.6-3.7)	3.25(2.60-4.00)	0.995	0.746	2.9(2.38-4.63)	2.3(1.8-3.55)	2.85(2.275-3.75)	0.202	0.686	0.069
HbA1c (%)	5.50(5.15-5.85)	5.5(5.18-5.73)	5.83(5.43-5.98)	0.946	0.404	7.3(5.90-8.70)	7.7(6.575-10.18)	8.1(7.35-9.175)	0.544	0.367	**<0.0001**
Fasting insulin (mU/L)	5.56(5.04-11.51)	6.08(4.99-15.67)	17.38(7.32-34.58)	0.448	0.015	13.37(4.08-30.63)	10.37(6.277-17.16)	17.15(7.909-43.42)	0.735	0.48	**0.008**

HOMA-IR	1.31(1.00-2.53)	1.351(1.16-3.61)	4.09(1.89-10.91)	0.419	**0.01**	5.53(1.11-7.49)	3.03(2.59-6.01)	5.74(3.078-17.69)	0.735	0.377	**<0.0001**
SRA mRNA(Fold level)	1.74(1.59-1.85)	1.822(1.23-2.20)	2.03(1.75-2.57)	0.489	**0.015**	2.51(1.26-3.01)	1.69(1.24-1.90)	1.814(1.35-2.567)	0.296	0.419	0.373

BMI, body mass index; WHR, Waist/Hip ratio; HDL, high-density lipoprotein; LDL, low-density lipoprotein; HbA1c, glycated hemoglobin; SD, standard deviation

Supplementary Table S2. List of TaqMan gene expression assays

Gene Name	Assay ID	Gene Name	Assay ID
**SRA1**	Hs00398296_g1	**NF-κB**	Hs00765730_m1
**TLR2**	Hs01872448_s1	**MyD88**	Hs01573837_g1
**TLR3**	Hs01551078_m1	**IRAK1**	Hs01018347_m1
**TLR4**	Hs00152939_m1	**TRAF6**	Hs00371512_g1
**TLR5**	Hs01920773_s1	**IRF3**	Hs01547283_m1
**TLR7**	Hs01933259_s1	**IRF4**	Hs01056533_m1
**TLR8**	Hs00152972_m1	**IRF5**	Hs00158114_m1
**TLR9**	Hs00370913_s1	**GAPDH**	Hs03929097_g1
**TLR10**	Hs01935337_s1		

Supplementary Figure S1

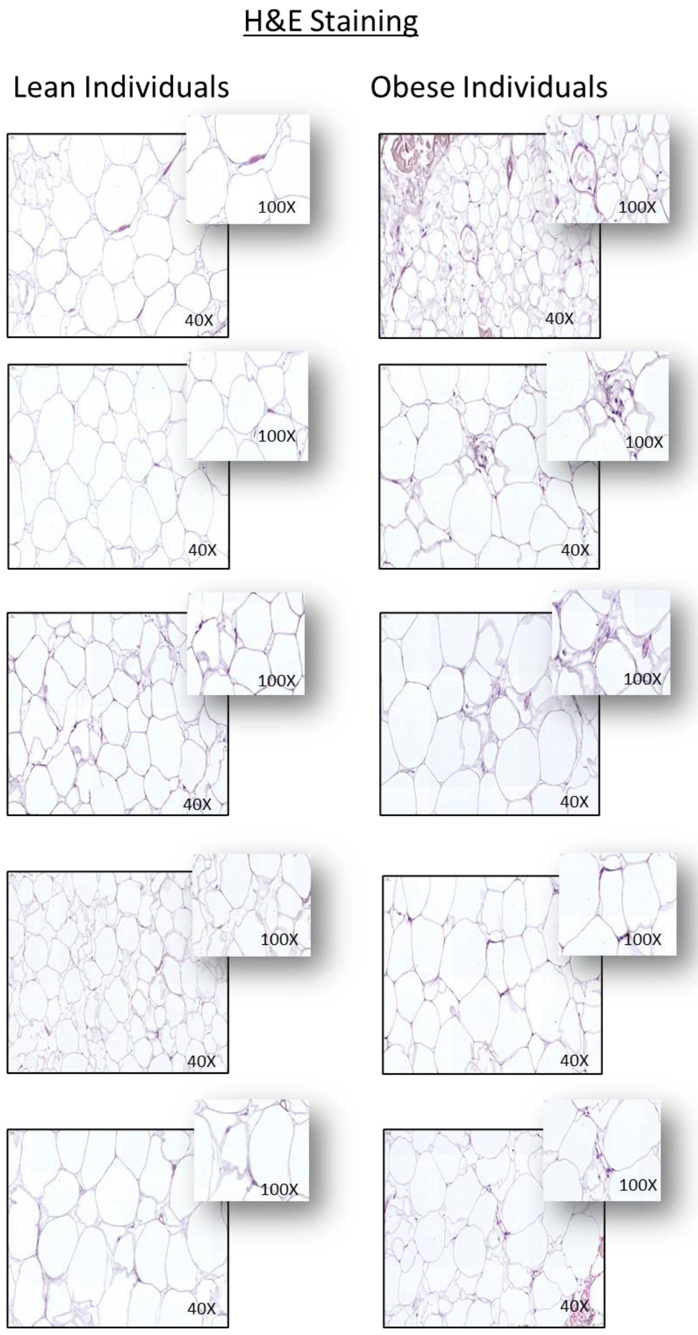

H&E Methodology:

For H&E staining, human adipose tissue samples were formalin-fixed, paraffin-embedded, and sectioned at 4 μm. For hematoxylin and eosin (H&E) staining, sections were deparaffinized and dehydrated with xylenes and ethanol. Briefly, slides were stained with hematoxylin, washed with water and 95% ethanol, and stained with eosin for 30 min. Sections were then incubated with ethanol and xylene, and mounted with mounting medium.
